# Cooperative P-Wave Velocity Measurement with Full Waveform Moment Tensor Inversion in Transversely Anisotropic Media

**DOI:** 10.3390/s22051935

**Published:** 2022-03-01

**Authors:** Ju Ma, Shuang Wu, Yuan Zhao, Guoyan Zhao

**Affiliations:** School of Resources and Safety Engineering, Central South University, Changsha 410083, China; majucsu@csu.edu.cn (J.M.); 215512102@csu.edu.cn (S.W.); gyzhao@csu.edu.cn (G.Z.)

**Keywords:** moment tensor inversion, tomography, full waveform, transversely anisotropic

## Abstract

Precise stochastic approaches to quantitatively calculate the source uncertainties offers the opportunity to eliminate the influence of anisotropy on moment tensor inversion. The effects of ignoring anisotropy were tested by using homogeneous Green’s functions. Results indicate the influence of anisotropy and noise on fault plane rotation is very small for a pure shear source whether it is restricted to double couple solution or full moment tensor solution. Green’s functions with different prior rough anisotropy information were tested, indicating that the complex source is more sensitive to velocity models than the pure shear source and the fault plane rotation caused by full moment tensor solution is larger than the pure double couple solution. Collaborative P-wave velocity inversion with active measurements and passive acoustic emission data using the fast-marching method were conducted, and new Green’s functions established based on the tomography results. The resolved fault plane solution rotated only 3.5° when using the new Green’s functions, but the presence of spurious isotropic and compensated linear vector dipole components was not completely eliminated. It is concluded that the cooperative inversion is capable of greatly improving the accuracy of the fault plane solutions and reducing the spurious components in the full moment tensor solution.

## 1. Introduction

Moment tensor inversion is an extremely advantageous tool which links the station observations to source models. The seismic moment tensor describes the event magnitude, the rupture type (e.g., shear, tensile), and the orientations of fractures by a matrix of nine force couples. Great progress has been made in moment tensor interpreting for ruptures at different scales. For example, Materna et al. (2019), Buijze et al. (2019), and Hensch et al. (2019) analyzed earthquake nucleation and the rupture process [1,2,3]. They successfully identified artificial nuclear explosions from natural earthquakes using moment tensor inversion. This technique is increasingly used for recognizing non-double couple earthquakes [4,5,6,7], discriminating volcanic events and hydraulic-fracturing induced earthquakes [8,9,10,11,12]. Besides, moment tensor inversion is applied for illuminating faulting complexities [13,14,15,16]. For example, Dublanchet et al. (2015) inferred fault mechanical conditions from the source parameters of a complex microseismic multiplet in Corinth rift, Greece [1,2,3]. In the past three years, seismic moment tensors have been adopted to analyze spatial and temporal stress variations through stress tensor evaluation [17,18,19,20] and to predict ground motions considering focal mechanisms [21,22,23,24]. For example, Hardebeck and Okada (2018), obtained temporal stress changes caused by earthquakes employing moment tensor inversion [1,2,3]. Ma et al. (2019) predicted ground motions induced by mining seismic events with different focal mechanisms [1,2,3].

Seismic moment tensor solutions are traditionally determined using 1-D, radially symmetric models [25,26]. This works well for global earthquakes with magnitudes larger than 5.5. However, the rock media is heterogeneous, with heterogeneities caused by the presence of ore bodies, dykes, and huge excavations in mining environments. The 3-D effects become severe and unneglected for moderate and micro-earthquakes with higher frequency waveforms in regional distances [27,28]. Due to the 3-D sensor network and the anisotropic velocity model, the ray paths from the source to the sensors are dramatically complicated. Oversimplified velocity models affect the accuracy of moment tensor inversion in varying degrees [29,30,31].

Sileny and Vavryčuk (2002) first mentioned that the compensated linear-vector dipole (CLVD) and the isotropic (ISO) components of the full moment tensor (FMT) will be overestimated for pure-shear sources when inverting events with waveforms recorded in anisotropic structures but assuming isotropy [32]. This finding was also confirmed by Hingee et al. (2011) [33]. Their researches claimed that inversions using 1-D Green’s functions cannot retrieve the correct source parameters generated by realistic 3-D model. Grechka (2020) explained that the reason for this phenomenon is the combination of anisotropy and orthorhombic focal regions, responsible for the deviations from double couples (DC) [34]. Menke and Russell (2020) also clarified that the intensities of the ISO and CLVD components are functions of the deviations of the medium isotropy [35]. Since the observations often contain noise, Jechumtálová and Bulant (2014) compared the accuracy of moment tensor inversion under the dual influences of anisotropy and noise contamination [36]. They demonstrated that coarse Green’s functions (even 1-D) may be sufficient when a completely high-quality dataset is available, but using exact 3-D Green’s functions enables the use of fewer data or data of lower quality to access reasonable results. To further understand the effect of anisotropy on moment tensor inversion in different scales (earthquakes, microseismic events, and acoustic emissions), Vavryčuk (2018) systematically studied the errors imported when anisotropy is neglected [37].

Generally, there are three ways to reduce the 3-D effects. The most direct way is to invert the tomographic image as accurately as possible, using the 3-D velocity structure model for moment tensor inversion [28,38,39,40]. The second way is stick to 1-D Green’s functions, but carry out the inversion in frequency domain [41,42]. With the advantage of lower dependency on the alignment of the observed seismograms, this method greatly reduced the impact of poorly modeling velocity structures [25]. The third way is to use stochastic approaches to quantitatively calculate the uncertainties of the full moment tensor (ISO and non-DC components) when using a simplified velocity model, e.g., by perturbing the dataset (e.g., randomly removing a certain partial of the available traces) to derive the probability density distribution of the moment tensors and to infer a likelihood interpretation [43,44].

The above methods have their own advantages and applicable conditions (e.g., only in medium-scale and large-scale scenarios). For the very high frequency and very small-scale acoustic emission data, there is no relevant attempt at present. Considering the inherent characteristics of acoustic emission data, such as the large amount of datasets and the nonnegligible anisotropy of rock mass, we consider combining the above three ways to adapt to the focal mechanism inversion of microseismic/acoustic emission events on a meter scale. This motived us to cooperate velocity tomography with full waveform moment tensor inversion in anisotropic media (transverse isotropy). The 3-D, anisotropic P-wave velocity image is inverted by the quasi-Newton method based on active measurements and passive microseismic observations. The moment tensor results are determined with a full waveform, stochastic boost method.

## 2. Method

### 2.1. Moment Tensor Inversion Algorithm

We employed a joint inversion scheme that estimates the moment tensors from a combination of seismic waveforms and waveform attributes. The algorithm explores the full model space and maps model parameter trade-offs through a Bayesian bootstrap-based stochastic optimization [45]. Instead of simulating the sampling distribution of a statistic estimating a parameter, the Bayesian bootstrap simulates the posterior distribution of the parameter [46]. Multiple objective functions (the misfit between observed and synthetic data) are explored in parallel as individual bootstrap chains. Meanwhile, an individual high-score list is maintained for each bootstrap chain and updated after each iteration when all objective functions are evaluated for a candidate model. Bootstrap chains converging to different areas of the model space represent the uncertainty of the models with respect to errors in the data. From the combination of results from all bootstrap chains’ high-score lists, the current best and mean solutions can be retrieved [47]. Stochastic analysis is widely used for mathematical models of systems and phenomena that appear to vary in a random manner. In areas relevant to this study, successful applications include the reliability assessment of shear wave velocity [48], nonlinear ground response analysis in cities [49], and deep shear wave velocity profiles with estimates of uncertainty in the complex interbedded geology [50].

### 2.2. Tomography Algorithm

**Figure 1:**
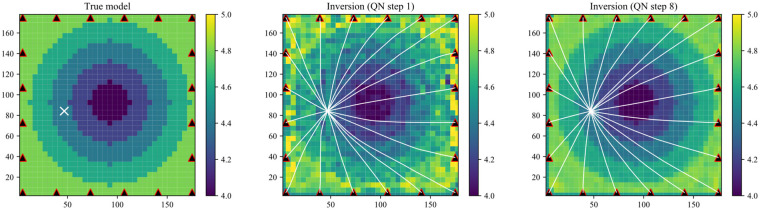
Synthetic example of an anisotropic model inversion.

Tomography inversion involves solving the physical relationship between the velocity and anisotropy (***E***) structure as well as the measured arrival times. Following the fast-marching algorithm, we defined the velocity and anisotropy structures along a regular orthonormal grid with relatively coarse spacing. The horizontal P-wave velocity (***V_h_***) structure is solved using the fast-marching method by tracing rays within a linearly refined grid. The iteration starts with the sensor locations, the prior source locations, the arrival times from surveys and sources, the prior ***V_h_*** model, and the prior ***E*** model. In order to limit the iteration range and increase the calculation speed, the grid refinement factor, the quasi-Newton step size, the maximum number of iterations, and the standard deviations on ***V_h_*** and ***E***, arrival time picks, source locations, and source origin times need to be set in advance. Figure 1 shows a synthetic example of an anisotropic model inversion. The white cross in Figure 1 shows the position of the event, and the red triangles shows the sensors. The left plot in Figure 1 shows the true model, based on which we generated the synthetic arrival times. The model was divided into a 36 × 36 coarse grid spacing. We used an isotropic model with the average velocity of the true velocity structure as the prior model. The middle plot in Figure 1 shows the inversion at the first quasi-Newton iteration. The right plot shows the tomography results at the 8th iteration step. The white lines indicate the rays from the source to the sensors at the corresponding resolved velocity model. The rays were traced by a refined grid with a factor of 10.

### 2.3. Configuration of the VTI Anisotropic Model

**Figure 2:**
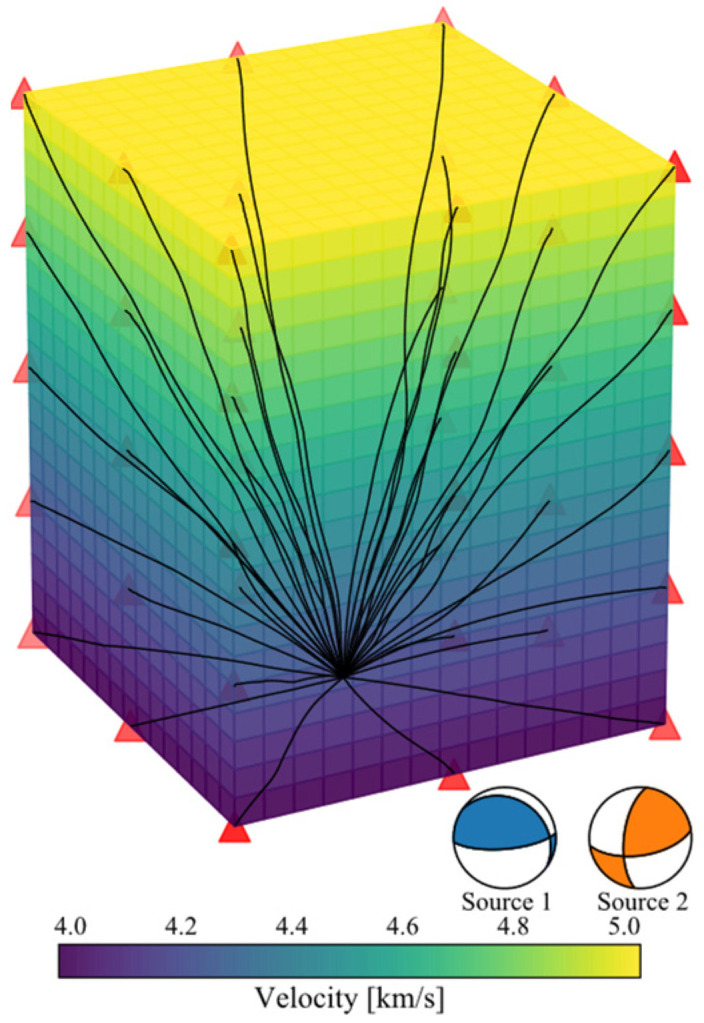
Configuration of the VTI anisotropic model, the source, and the sensor network. The source is located at the center of the bottom layer. The sensors are presented by red triangles. The black curves represent the ray path from the source to the sensor.

Stress and fracture usually generate anisotropies aligned with the compression axis. In practice, the scene of initially rockmass subjected to triaxial compression can be recognized as a VTI (vertically transversely isotropic) geometry, especially on meter scale or smaller scales (e.g., in laboratory experiment). The VTI anisotropic model we assumed is a 4.8 × 4.8 × 6.4 m cuboid with wave velocity increases uniformly from 4.0 m/s at the bottom to 5.0 m/s at the top (Figure 2). The source is located at the center of the bottom layer (depth of 6.4 m) with moment magnitude of −4.0. According to the Nyquist’s theory, the sampling frequency needs to be at least twice of the corner frequency. Since we focus on the events with a dominant frequency below 2500 Hz, the sampling frequency of the seismogram used during moment tensor inversion is 5000 Hz.

In this study, we assumed two source models. Source 1 is a pure shear source with strike of 85.0°, dip of 72.0°, and rake of 76.0°. Source 2 is a complex non-double couple source with ISO, DC, and CLVD of 52.1%, 8.3%, and 39.6% respectively. Their source configurations are listed in Table 1. We arranged a 3-D sensor network around the source, which is divided into five layers in the vertical direction, with an interval of 1.6 m between each layer. There are 8 sensors distributed with equal angular interval on each layer (Figure 2). These sensors are able to passively record acoustic emission signals, allowing active ultrasonic probing at repeated time intervals.

## 3. The Influence of Anisotropy on the Inversion of Focal Mechanism

In order to analyze the influence of anisotropy on the inversion of focal mechanism, we conducted two sets of experiments. The first set of experiments was performed without any prior anisotropy information. Homogeneous Green’s functions under increasing noise scales were used in the inversion. In the second set of experiments, we used simplified layered Green’s functions with inaccurate anisotropic prior information. The basic configurations of the Green’s functions used in these two experiments are the same: the sampling rate is 5000 Hz, the receiver delta is 1.6 m, the source delta is 0.1 m, and the distance delta is 0.1 m. The ratio of P-wave velocity to S-wave is 1.769 at same position. The Green’s functions were calculated by reflectivity-type wavenumber integration methods in cylindrical symmetry with receivers at the varying depth.

### 3.1. Inversion without Prior Anisotropy Information

The waveforms at the sensor positions based on the anisotropic model were generated (Section 2.3). Increasing noise levels with scales 3 × 10^−7^ and 6 × 10^−7^ were added to the noise free data. Homogeneous Green’s functions with P wave velocity of 4000 m/s, 4250 m/s, 4500 m/s, 4750 m/s, and 5000 m/s were built. The moment tensor inversions were performed in two scenarios, one to limit the solution to double couple and the other to solve the full moment tensor. The frequency band of acausal filter was set to 800–900 Hz. The relative to hypocenter origin time to be searched in the optimization is ±0.0005 s. The centroid location with respect to hypocenter origin is ±0.1 m.

**Figure 3:**
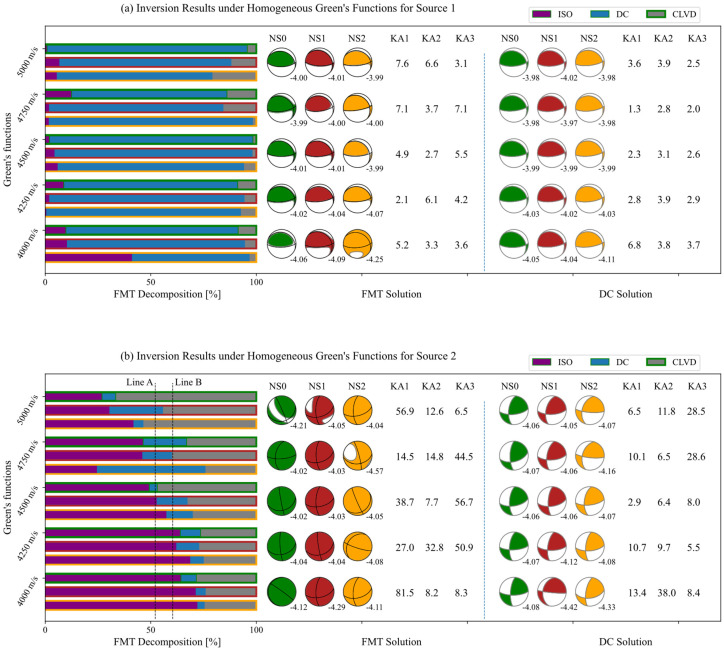
Inversion under homogeneous Green’s function with increasing noise levels for the two sources.

The inversion was performed in the time domain and the results are shown in Figure 3. The inversion was restricted to DC solution and FMT solution separately. The NS0, NS1, and NS2 indicate noise free, noise scale 3 × 10^−7^, and scale 6 × 10^−7^ respectively. The KA1, KA2, and KA3 indicate the Kagan angel of the solution to the real fault planes under corresponding noise levels. The dotted line A and line B in the FMT decomposition indicate the real ratio of the ISO component and the DC component. The black solid lines in the FMT beachballs show the decomposed DC solution. The numbers in the lower right corner of the beachball indicate the inverted moment magnitude.

For the pure shear source (Source 1, Figure 3a), it does not seem the case that the greater the noise, the greater the rotation of the resolved fault plane. The influence of the 3D wave velocity anisotropy and noise on fault plane rotation is very small whether it is restricted to DC solution or FMT solution. Relatively speaking, the greater the difference between the velocity of the isotropic model and the actual velocity at the source, the greater the rotation of the fault plane. For example, the Kagan angle is 2.1° when using isotropic Green’s function with P-wave velocity of 4250 m/s, while it is 7.6° in the model of 5000 m/s. Noise and wave velocity anisotropy both increase the non-DC component. The influence of noise is greater than that of the anisotropy. The largest non-DC component occurs in the 4000 m/s isotropic model with noise scale of 6 × 10^−7^, where the ratio of ISO component is 42% and the ratio of CLVD component is 4%. The pure DC solution is not consistent with the DC component in FMT. No matter the noise or the wave velocity anisotropy, it has no major impact on the inverted magnitude.

It is noticed that for the complex source (Source 2, Figure 3b), noise and anisotropy have varying impacts on the moment tensor solution, not only causing the nodal plane to be rotated, but also causing the ratio of each component to change. It can be seen that the greater the velocity difference between the homogeneous model and the actual structure, the smaller the ISO component resolved. For example, the ISO component reduced by 23% when using isotropic Green’s function with P-wave velocity of 5000 m/s, but increased by 20% when using homogeneous model with 4000 m/s. When the velocity of the used Green’s function differs greatly from the actual value at the source, the noise has a greater impact on the proportion of the non-DC component. For example, the DC component increased by three times when using velocity of 5000 m/s under noise scale of 3 × 10^−7^ and increased by 5 times with velocity of 4750 m/s under noise scale 6 × 10^−7^. The inaccuracy of the velocity model leads to large errors in the solved fault plane, and even leads to a maximum rotation of 81.5°. We noticed that the influence of the isotropic velocity model and noise on the complex seismic source (the Source 2) is significantly greater than that of the pure shear source (the Source 1). Whether it is pure shear source or complex non-double couple source, the fault plane solution from pure DC inversion is more accurate then that from the FMT inversion. For example, the minimum rotation of the DC solution is 2.9° for the Source 2 when noise free data are used, while the corresponding FMT is rotated by 38.7° under the same conditions. We even conclude that in case of inaccurate velocity models, the moment tensor solution results of complex seismic sources are unreliable, especially for high-frequency events on a small scale.

### 3.2. Inversion with Prior Anisotropy Information

**Figure 4:**
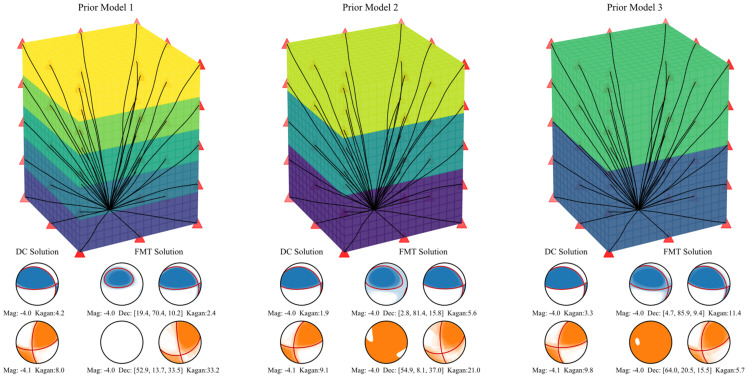
The inversion results under Green’s functions with prior anisotropy information.

In actual engineering, we more or less know some basic prior anisotropy information (e.g., where the wave velocity is higher or lower) about the area of interest. To this end, we established three layered velocity models after premeditating different prior anisotropy information. The three models (Figure 4) consider five, three, and two isotropic layers, respectively. The velocity of each layer corresponds to the average velocity of the corresponding geometry in the actual model (Figure 2). The velocity for the five layers in Prior Model 1 is 4.2 km/s, 4.4 km/s, 4.6 km/s, 4.8 km/s, and 5.0 km/s, from bottom to top, respectively. The velocity for the three layers in Prior Model 2 is 4.25 km/s, 4.5 km/s, and 4.75 km/s, from bottom to top, respectively. The velocity for the two layers in Prior Model 3 is 4.25 km/s for the bottom and 4.75 km/s for the top. The difference between these three prior models and the actual model increases in turn (Figure 4). The waveform data used in this test were generated by the actual model without adding any noise. The inversion configuration parameters were set the same as that in the Section 3.1. The inversion results are shown in Figure 4. The beachballs in cyan color correspond to Source 1, and the ones in orange color correspond to Source 2. The left beachball shows the pure DC solution, the middle one shows the FMT solution, and the right one shows the DC component decomposed from the FMT solution. The moment magnitude, the fault plane rotation (Kagan angle), and the FMT decomposition (in format (ISO, DC, CLVD)) are listed below the beachballs.

It is obviously noticed that when using the inhomogeneous medium model with prior anisotropy information, the pure DC solution is different from the DC component decomposed from the FMT solution. The fault plane rotation caused by FMT solution is larger than the pure DC solution. For example, The Kagan angle is 11.4° for the DC component form FMT solution when Prior Model 3 is used for the Source 1, but it is only 3.3° for the pure DC solution. When Prior Model 1 is used for the Source 2, the fault plane rotated 33.2° for the FMT solution, but only rotated 8.0° when restricted to pure DC solution. The above results also indicate that the complex source is more sensitive to velocity models than the pure shear source. From the comparison of the three prior models, the increase of the difference from the real model does not necessarily lead to the increase of non-DC components. In other words, the applied probability inversion method can effectively reduce the error that may be caused by the inaccuracy of the velocity model.

Comparing Figure 3 and Figure 4, we can see that the solutions from Green’s functions with prior anisotropy information is much better than that without any prior anisotropy information. For Source1, the maximum Kagan angle of DC solution is 4.2° (using Prior Model 1), and it is 7.6° when using velocity model without any prior anisotropy information (homogeneous Green’s function with P wave velocity of 5000 m/s). However, even if the prior models are used, the calculated FMT solutions still have an unignorable non-DC component for Source 1. There is not much improvement compared with the inversions when using homogeneous models. However, basically correct FMT solutions were obtained for Source 2 when using Prior Model 1 and Prior Model 2. In comparison between the prior models, generally, the more accurate the velocity model, the more reliable the inversion result. That is why accurate tomography is required.

## 4. Tomography

**Figure 5:**
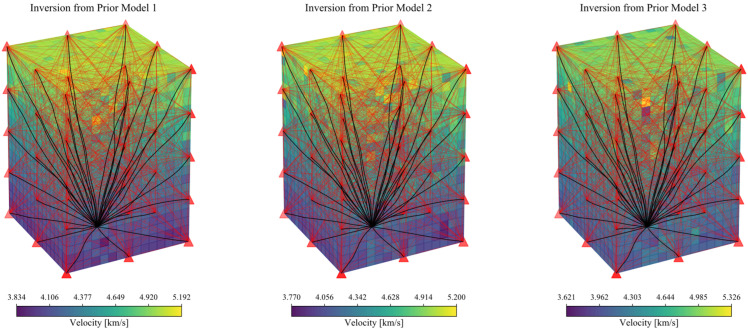
Tomography results with the three prior models. The red curves indicate the rays of active measurements which providing paths with definitely source positions. The black curves represent the ray path from the source to the sensors. The red triangles indicate the sensors.

The coverage of ray paths for single event is not dense enough to constrain the full 3D velocity structure. On the one hand, we used the active probing function of the sensors to increase paths with definitely source positions. On the other hand, we randomly generated 600 events and calculated the arrival times of each event to the sensors using the fast-marching algorithm. Consequently, we conducted collaborative velocity inversion with active measurements and passive acoustic emission data. Except for the difference of the prior model, all the configurations used in the three anisotropic media inversions are the same. The inversion parameters used for the velocity imaging are listed in Table 2. Essentially, the correlation length provides a smoothness of the velocity structure, preventing (statistically) heterogeneities over length scales smaller than it. That is, the roughness of the inversion result is controlled by the correlation length. Comprehensive considering the grid spacing (80 mm) and the grid refinement factor (10), the correlation length is selected as 5 mm. The inversion results are shown in Figure 5.

**Figure 6:**
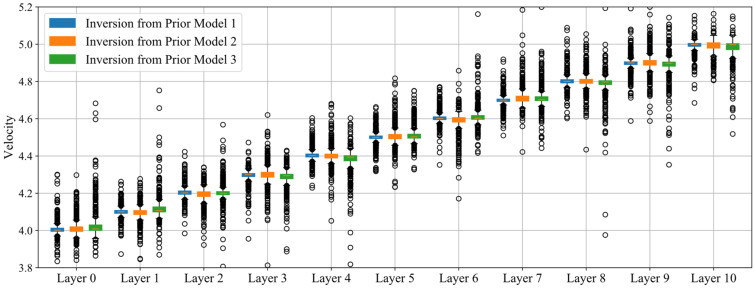
Statistics of the solution distribution in each layer (every 0.64 m, See Table 3) with the three prior models. The unit for the velocity is km/s.

It can be seen from Figure 5 that the inversion results all match the actual situation. Moreover, the topper the structure, the greater the velocity. The tomography result of the inversion from Prior Model 1 is the most accurate, and the overall resolved velocities are concentrated with 3.834 km/s to 5.192 km/s. As the prior model becomes rougher, the inversion results become more discrete and the overall error of the inversion result grows. In order to analyze the accuracy of the resolved velocity at each node, we divided the model every 0.64 m in the vertical direction, and then counted the solution distribution by layer. The statistical results are shown in Figure 6. The layer ranges (m) specified for Figure 6 are listed in Table 3 and the standard deviation in different layers are listed in Table 4.

From the distribution of the standard deviation, the closer to the top layer (farther from the source), the more discrete the results when under a specific priority model. From the comparison of the three prior models, the discreteness of Model 3 is greater than that of Model 2, and the results from Model 2 are greater than those of Model 1. It can be noticed from the statistical results that although the inversion results are becoming more and more discrete from Prior Model 1 to Model 3, the average value in each layer of the models is almost the same as the actual value. We can be sure that such tomography results are acceptable.

## 5. MT Reinversion Results and Discussion

**Figure 7:**
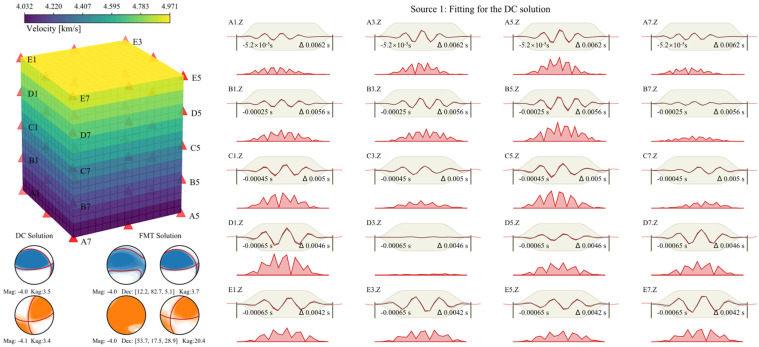
The new VTI Green’s function, the inversion results of the two sources, and the fitting for the DC solution for the Source 1.

Based on the velocity imaging results (Figure 5), we used the average velocity of each layer (every 0.64 m in vertical) as the inverted VTI model, and established the new Green’s function (Figure 7). According to the inversion method mentioned in Section 2.1, we recalculated the focal mechanism of the two events (Figure 7). The orientation of the focal mechanism appears to be robustly retrieved. The resolved fault plane solution rotated only 3.5° for the Source1 and rotated merely 3.4° for the Source 2. This is a huge improvement for Source 2 compared with the result when using homogeneous models (Figure 3) and the result when using inaccurate anisotropy models (Figure 4). We can conclude that, for complex sources, in order to ensure the accuracy of the resolved focal mechanism, the velocity model needs to be as accurate as possible, while pure shear sources (such as fault slips) have relatively low requirements for the velocity models.

Even when the velocity model is relatively accurate, the non-DC component of Source 1 still increased 17.3%. The neglect of anisotropy results in the presence of spurious ISO and CLVD components indeed. However, even if anisotropy is considered, if it is not accurate enough, this effect is inevitable. It is easy to mislead the type of event (e.g., fault slip or collapse of rock due to mining activity). Therefore, it is necessary to comprehensively predict event types before the inversion (e.g., through location analysis).

The fundamental reason for the deviation of the focal mechanism is that the ray paths are sensitive to the velocity model. While the ray paths are straight lines in the homogeneous velocity model, the real ray paths may be curved in heterogeneous structures. Kühn and Vavryčuk (2013) reported that a complicated 3D velocity model with strong velocity contrasts can distort not only the P-wave polarity pattern, but also the P-wave amplitudes [27]. Consequently, the positions of P-wave arrival and the first onset polarities can be remarkably different due to ray bending. For the simplicity of the anisotropic VTI model (Figure 7), the travel-time difference increases with the take-off angle of the seismic rays. The slightest deviation in the 3D velocity may have a significant impact on the fitting of the full waveforms, especially for the non-shear sources.

## 6. Conclusions

Small changes in velocity may lead to remarkable changes in ray paths and inevitably lead to unreliable moment tensor results. Now, with dense seismic arrays and advanced imaging methods, 3D rock velocity imaging has become more accurate. In addition to improving the accuracy of the velocity model, stochastic approaches were adopted to quantitatively calculate the source uncertainties to significantly eliminate the influence of anisotropy on moment tensor inversion.

To clarify the impact of transverse anisotropy on high-frequency events at the scale of meters, we considered an anisotropic medium with 20 levels of anisotropy ranging from 4 to 5 km/s in P waves and carried out three synthetic tests: (1) directly using homogeneous Green’s functions, (2) using Green’s functions with different prior rough anisotropy, and (3) conducting collaborative velocity inversion using the fast-marching method, establishing new Green’s functions based on the tomography results. The main findings including:

(1) The influence of anisotropy and noise on fault plane rotation is very small for a pure shear source whether it is restricted to double couple solution or full moment tensor solution. However, it may have varying impacts on the moment tensor solution for the complex source, not only causing the nodal plane to be rotated, but also causing the ratio of each component to change.

(2) The velocity model needs to be as accurate as possible to ensure the accuracy of the resolved focal mechanism for complex sources, while pure shear sources have relatively low requirements for the velocity models. When an accurate 3D velocity model cannot be obtained, providing the necessary prior information about the velocity distribution is of great help to the results.

(3) The cooperative inversion is capable of greatly improving the accuracy of the fault plane solutions and reducing the spurious components in the full moment tensor solution. This method can continue to be tested and extended at similar meter scales.

## Figures and Tables

**Table 1:** The moment tensor decompositions and fault plane solutions of the two sources.

	ISO [%]	DC [%]	CLVD [%]	Strike [°]	Dip [°]	Rake [°]	Type
Source 1	0.0	100.0	0.0	85.0	72.0	76.0	Pure shear source
Source 2	52.1	8.3	39.6	81.1	61.5	149.8	Complex non-double couple source

**Table 2:** Inversion parameters used for the velocity tomography.

Grid Spacing [mm]	Grid Refinement Factor	Step Size	Correlation Length [mm]	SD * on Location [mm]	SD on *t*_0_ [µs]	SD on Event Picks [µs]	SD on Survey Picks [µs]	SD on *ln* (*V_h_*)	SD on *E*
80	10	1.0	5	20/20/20	0.2	0.01	0.01	0.1	0.1

* SD is short for standard deviation.

**Table 3:** Layer ranges (m) specified for Figure 6. The direction of the z-axis is from bottom to top.

Layer 0	Layer 1	Layer 2	Layer 3	Layer 4	Layer 5	Layer 6	Layer 7	Layer 8	Layer 9	Layer 10
0–0.64	0.64–1.28	1.28–1.92	1.92–2.56	2.56–3.2	3.2–3.84	3.84–4.48	4.84–4.48	4.48–5.12	5.12–5.76	5.76–6.4

**Table 4:** Standard deviation in different layers.

	Layer 0	Layer 1	Layer 2	Layer 3	Layer 4	Layer 5	Layer 6	Layer 7	Layer 8	Layer 9	Layer 10
Prior Modle 1	0.042	0.031	0.035	0.038	0.036	0.037	0.035	0.036	0.044	0.044	0.046
Prior Model 2	0.047	0.041	0.043	0.058	0.053	0.054	0.056	0.055	0.053	0.055	0.048
Prior Model 3	0.082	0.072	0.060	0.053	0.063	0.047	0.060	0.057	0.076	0.075	0.077

## Data Availability

The waveform and station data are publicly available (Ma, Ju (2020), Dataset, Dryad, Dataset, https://doi.org/10.5061/dryad.fqz612jrs; accessed on 12 January 2022).

## References

[1] Materna K., Wei S., Wang X., Heng L., Wang T., Chen W., Salman R., Bürgmann R. (2019). Source characteristics of the 2017 Mw6.4 Moijabana, Botswana earthquake, a rare lower-crustal event within an ancient zone of weakness. Earth Planet. Sci. Lett..

[2] Buijze L., Bogert P.A.J., Wassing B.B.T., Orlic B. (2019). Nucleation and Arrest of Dynamic Rupture Induced by Reservoir Depletion. J. Geophys. Res. Solid Earth.

[3] Hensch M., Dahm T., Ritter J., Heimann S., Schmidt B., Stange S., Lehmann K. (2019). Deep low-frequency earthquakes reveal ongoing magmatic recharge beneath Laacher See Volcano (Eifel, Germany). Geophys. J. Int..

[4] Vavryčuk V., Adamová P. (2020). Non-Double-Couple Moment Tensors of Earthquakes Calculated Using Empirical Green’s Functions. Seismol. Res. Lett..

[5] Wang R., Gu Y.J., Schultz R., Chen Y. (2018). Faults and Non-Double-Couple Components for Induced Earthquakes. Geophys. Res. Lett..

[6] Vavryčuk V. (2011). Tensile earthquakes: Theory, modeling, and inversion. J. Geophys. Res..

[7] Šílený J., Hill D.P., Eisner L., Cornet F.H. (2009). Non–double-couple mechanisms of microearthquakes induced by hydraulic fracturing. J. Geophys. Res..

[8] Wang T., Shi Q., Nikkhoo M., Wei S., Barbot S., Dreger D., Bürgmann R., Motagh M., Chen Q.-F. (2018). The rise, collapse, and compaction of Mt. Mantap from the 3 September 2017 North Korean nuclear test. Science.

[9] Grigoli F., Cesca S., Rinaldi A.P., Manconi A., López-Comino J.A., Clinton J.F., Westaway R., Cauzzi C., Dahm T., Wiemer S. (2018). The November 2017 M w 5.5 Pohang earthquake: A possible case of induced seismicity in South Korea. Science.

[10] Alvizuri C., Silwal V., Krischer L., Tape C. (2018). Estimation of Full Moment Tensors, Including Uncertainties, for Nuclear Explosions, Volcanic Events, and Earthquakes. J. Geophys. Res. Solid Earth.

[11] Kim K.-H., Ree J.-H., Kim Y., Kim S., Kang S.Y., Seo W. (2018). Assessing whether the 2017 M w 5.4 Pohang earthquake in South Korea was an induced event. Science.

[12] Ma J., Dong L., Zhao G., Li X. (2018). Discrimination of seismic sources in an underground mine using full waveform inversion. Int. J. Rock Mech. Min. Sci..

[13] He X., Ni S., Zhang P., Freymueller J. (2018). The 1 May 2017 British Columbia-Alaska Earthquake Doublet and Implication for Complexity Near Southern End of Denali Fault System. Geophys. Res. Lett..

[14] Shelly D.R. (2015). Complexity of the deep San Andreas Fault zone defined by cascading tremor. Nat. Geosci..

[15] Tape C., Holtkamp S., Silwal V., Hawthorne J., Kaneko Y., Ampuero J.P., Ji C., Ruppert N., Smith K., West M.E. (2018). Earthquake nucleation and fault slip complexity in the lower crust of central Alaska. Nat. Geosci..

[16] Dublanchet P., Godano M., Bernard P. (2015). Inferring fault mechanical conditions from the source parameters of a complex microseismic multiplet in the Corinth rift, Greece. J. Geophys. Res. Solid Earth.

[17] Zhang H., Eaton D.W., Rodriguez G., Jia S.Q. (2019). Source-Mechanism Analysis and Stress Inversion for Hydraulic-Fracturing-Induced Event Sequences near Fox Creek, Alberta. Bull. Seismol. Soc. Am..

[18] Chang Y., Warren L.M., Zhu L., Prieto G.A. (2019). Earthquake Focal Mechanisms and Stress Field for the Intermediate-Depth Cauca Cluster, Colombia. J. Geophys. Res. Solid Earth.

[19] Hardebeck J.L., Okada T. (2018). Temporal Stress Changes Caused by Earthquakes: A Review. J. Geophys. Res. Solid Earth.

[20] Yu Z., Zhao D., Li J., Huang Z., Nishizono Y., Inakura H. (2019). Stress Field in the 2016 Kumamoto Earthquake (M 7.3) Area. J. Geophys. Res. Solid Earth.

[21] Ma J., Dong L., Zhao G., Li X. (2019). Qualitative Method and Case Study for Ground Vibration of Tunnels Induced by Fault-Slip in Underground Mine. Rock Mech. Rock Eng..

[22] Ma J., Dong L., Zhao G., Li X. (2019). Ground motions induced by mining seismic events with different focal mechanisms. Int. J. Rock Mech. Min. Sci..

[23] Dahm T., Heimann S., Funke S., Wendt S., Rappsilber I., Bindi D., Plenefisch T., Cotton F. (2018). Seismicity in the block mountains between Halle and Leipzig, Central Germany: Centroid moment tensors, ground motion simulation, and felt intensities of two M ≈ 3 earthquakes in 2015 and 2017. J. Seismol..

[24] Suzuki W., Aoi S., Kunugi T., Kubo H., Morikawa N., Nakamura H., Kimura T., Fujiwara H. (2017). Strong motions observed by K-NET and KiK-net during the 2016 Kumamoto earthquake sequence. Earth Planets Space.

[25] Ma J., Dineva S., Cesca S., Heimann S. (2018). Moment tensor inversion with three-dimensional sensor configuration of mining induced seismicity (Kiruna mine, Sweden). Geophys. J. Int..

[26] Sen A.T., Cesca S., Bischoff M., Meier T., Dahm T. (2013). Automated full moment tensor inversion of coal mining-induced seismicity. Geophys. J. Int..

[27] Kühn D., Vavryčuk V. (2013). Determination of full moment tensors of microseismic events in a very heterogeneous mining environment. Tectonophysics.

[28] Zhou L., Zhang W., Shen Y., Chen X., Zhang J. (2016). Location and moment tensor inversion of small earthquakes using 3D Green’s functions in models with rugged topography: Application to the Longmenshan fault zone. Earthq. Sci..

[29] Leaney W.S. (2014). Microseismic Source Inversion in Anisotropic Media.

[30] Shi P., Angus D., Nowacki A., Yuan S., Wang Y. (2018). Microseismic Full Waveform Modeling in Anisotropic Media with Moment Tensor Implementation. Surv. Geophys..

[31] Boitz N., Reshetnikov A., Shapiro S.A. (2018). Visualizing effects of anisotropy on seismic moments and their potency-tensor isotropic equivalent. Geophysics.

[32] Šílený J., Vavryčuk V. (2002). Can unbiased source be retrieved from anisotropic waveforms by using an isotropic model of the medium?. Tectonophysics.

[33] Hingee M., Tkalčić H., Fichtner A., Sambridge M. (2011). Seismic moment tensor inversion using a 3-D structural model: Applications for the Australian region. Geophys. J. Int..

[34] Grechka V. (2020). Moment tensors of double-couple microseismic sources in anisotropic formations. Geophysics.

[35] Menke W., Russell J.B. (2020). Non-Double-Couple Components of the Moment Tensor in a Transversely Isotropic Medium. Bull. Seismol. Soc. Am..

[36] Jechumtálová Z., Bulant P. (2014). Effects of 1-D versus 3-D velocity models on moment tensor inversion in the Dobrá Voda area in the Little Carpathians region, Slovakia. J. Seismol..

[37] Vavryčuk V. (2018). Seismic Moment Tensors in Anisotropic Media: A Review. Moment Tensor Solut..

[38] Zhu L., Zhou X. (2016). Seismic moment tensor inversion using 3D velocity model and its application to the 2013 Lushan earthquake sequence. Phys. Chem. Earth, Parts A/B/C.

[39] Takemura S., Matsuzawa T., Kimura T., Tonegawa T., Shiomi K. (2018). Centroid Moment Tensor Inversion of Shallow Very Low Frequency Earthquakes Off the Kii Peninsula, Japan, Using a Three-Dimensional Velocity Structure Model. Geophys. Res. Lett..

[40] Newrkla K., Shiddiqi H.A., Jerkins A.E., Keers H., Ottemöller L. (2019). Implications of 3D Seismic Raytracing on Focal Mechanism Determination. Bull. Seismol. Soc. Am..

[41] Dahm T., Krüger F. (1999). Higher-degree moment tensor inversion using far-field broad-band recordings: Theory and evaluation of the method with application to the 1994 Bolivia deep earthquake. Geophys. J. Int..

[42] Cesca S., Buforn E., Dahm T. (2006). Amplitude spectra moment tensor inversion of shallow earthquakes in Spain. Geophys. J. Int..

[43] Cesca S., Rohr A., Dahm T. (2013). Discrimination of induced seismicity by full moment tensor inversion and decomposition. J. Seismol..

[44] Zhou J., Zhou Z., Zhao Y., Cai X. (2021). Global Wave Velocity Change Measurement of Rock Material by Full-Waveform Correlation. Sensors.

[45] Heimann S., Isken M., Kühn D., Sudhaus H., Steinberg A., Daout S., Cesca S., Bathke H., Dahm T. (2018). Grond: A Probabilistic Earthquake Source Inversion Framework.

[46] Rubin D.B. (1981). The Bayesian Bootstrap. Ann. Stat..

[47] Kühn D., Heimann S., Isken M.P., Ruigrok E., Dost B. (2020). Probabilistic Moment Tensor Inversion for Hydrocarbon-Induced Seismicity in the Groningen Gas Field, The Netherlands, Part 1: Testing. Bull. Seismol. Soc. Am..

[48] Johari A., Javadi A.A., Elmi M., Raei S. (2013). An analytical approach to reliability assessment of the shear wave velocity relationship. Sci. Iran..

[49] Johari A., Amjadi A.H., Heidari A. (2021). Stochastic nonlinear ground response analysis via a case study site in Shiraz city. Sci. Iran..

[50] Teague D., Cox B., Bradley B., Wotherspoon L. (2018). Development of deep shear wave velocity profiles with estimates of uncertainty in the complex interbedded geology of christchurch, New Zealand. Earthq. Spectra.

